# Does the Intensity of IGG4 Immunostaining Have a Correlation with the Clinical Presentation of Riedel's Thyroiditis?

**DOI:** 10.1155/2018/4101323

**Published:** 2018-04-16

**Authors:** C. A. Simões, M. R. Tavares, N. M. M. Andrade, T. M. Uehara, R. A. Dedivitis, C. R. Cernea

**Affiliations:** Department of Head and Neck Surgery, Hospital das Clínicas, School of Medicine, University of São Paulo, São Paulo, SP, Brazil

## Abstract

Riedel's thyroiditis (RT) represents one type of IgG4-related thyroid disease (IgG4RTD) and the diagnosis involves quantitative immunohistochemistry showing dense lymphoplasmacellular inflammatory infiltrate consisting of IgG4-positive plasma cells with storiform fibrosis and obliterative phlebitis. We report a case of RT with progressive enlargement of the anterior neck, severe dysphagia, odynophagia, and dyspnea. The patient underwent surgical decompression of the airway, protection tracheotomy, and gastrostomy for nutritional intake 6 months after first symptoms. Complete resolution occurred after surgical treatment combined with prednisolone. Immunostaining revealed IgG4-positive plasma cells 12/HPF (high-power field) and the IgG4/IgG ratio 25%, values that were disproportionate to the intensity of the patient's symptoms. As to this case and the few cases described and analyzed in the literature, our impression is that there is no relation between the intensity of symptoms in RT with the total number of IgG4-positive plasma cells and the IgG4/IgG ratio, but more studies are needed.

## 1. Introduction

The German surgeon Bernhard Moritz Carl Ludwig Riedel first described Riedel's thyroiditis in 1883, as a rare disease which changes the thyroid parenchyma texture into an extremely hard lesion with adhesion to the trachea and obstructive symptoms [1]. It is a chronic inflammatory condition which destroys the gland and affects the surrounding structures [1, 2]. The more evident etiology is autoimmune [1, 3, 4].

The differential diagnosis should consider malignant thyroid tumors [5], including the undifferentiated carcinoma. If performed by an experienced team, the fine needle aspirative biopsy (FNAB) suggests the diagnosis of Riedel's thyroiditis. However, a definitive diagnosis depends on an open incisional biopsy of the gland [6]. The conservative treatment is well established [5], whereas the surgical approach has both diagnostic and decompression roles.

Riedel's thyroiditis shows several features that justify its inclusion into the spectrum of IgG4 related disease (IgG4-RD). Foremost among them are the fibroinflammatory nature of the infiltrate, the presence of obliterative phlebitis, and its association with other forms of fibrosclerosis including sclerosing cholangitis, as well as retroperitoneal fibrosis, among others. One-third of patients with Riedel's thyroiditis develop fibrosing disorders in other organs. This inclusion was supported by the fact that RT showed elevated numbers of IgG4 positive plasma cells as well as the morphologic features of IgG4 related disease [7]. Glucocorticoids are the primary form of therapy in IgG4RD. However, their role in IgG4RTD needs to be evaluated [8, 9].

Today RT, the fibrosing variant of Hashimoto's thyroiditis, and a few patients of Graves' orbitopathy represent the types of IgG4-related thyroid disease (IgG4RTD). The diagnosis involves establishing high circulating levels of IgG4 > 135 mg/dL, increased serum IgG4 to IgG ratio of >8%, immunohistochemistry showing dense lymphoplasmacellular inflammatory infiltrate consisting of IgG4-positive plasma cells with storiform fibrosis and obliterative phlebitis, and increased IgG4 positive plasma cell > 10 cells per high-power field when at least three fields are evaluated and the IgG4/IgG ratio of RT is increased by varying degrees [7, 10].

One question not explained in the literature is the relation between the intensity of the thyroiditis, the total number of IgG4-positive plasma cells, and the IgG4/IgG ratio. Immunohistochemistry can show whether IgG-4 plasma cells are increased which could lead to fibrosis in other organs [11].

We present the case of an exuberant manifestation of the disease with dysphagia and dyspnea without a large quantitative manifestation in the immunohistochemical analysis.

## 2. Case Report

**Figure 1:**
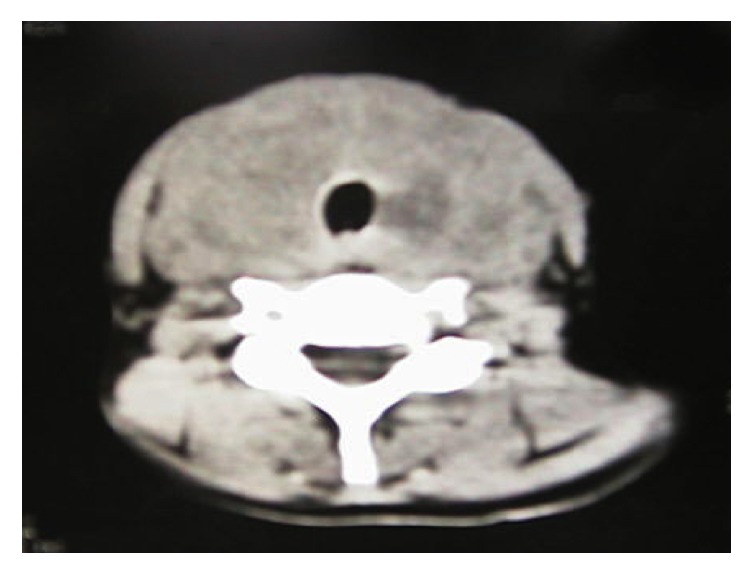
Axial computed tomography showing diffuse infiltrate with no precise boundaries around the thyroid.

**Figure 2:**
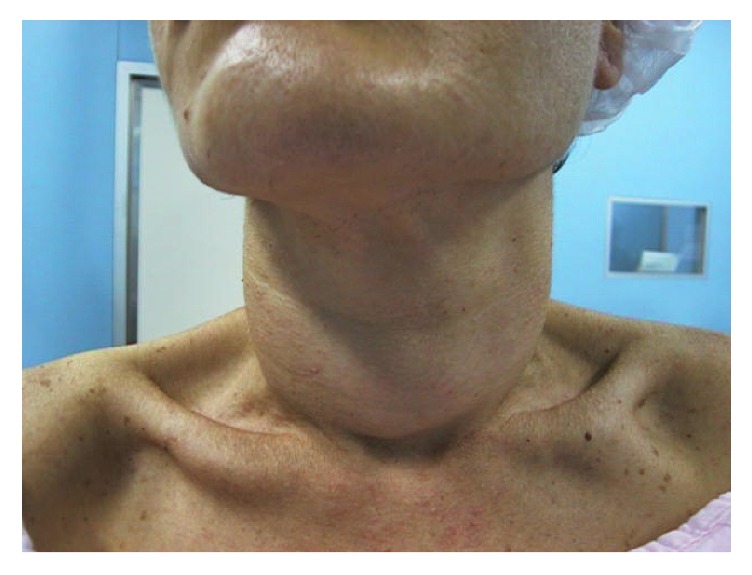
Increase of the anterior cervical volume during 4 months of evolution. Note the weight loss.

We describe a 40-year-old woman complaining of a progressive painless enlargement of the anterior neck, dyspnea, dysphagia, odynophagia, and weight loss of 7 Kg in 4 months. The thyroid gland was hard and the ultrasound exam calculated a volume of 70 g. The patient had hypothyroidism and was undergoing hormonal replacement. Fine needle aspiration was suggestive of Riedel's thyroiditis. The CT scan confirmed a large thyroid lesion with esophageal invasion and decreased tracheal lumen of about 50% (Figure 1). Bilateral vocal fold paresis was verified by means of a laryngoscopy. The dysphagia and dyspnea worsened progressively and the weight loss reached 21 Kg in 6 months after the first symptoms, so she needed a surgical approach for diagnosis and treatment (Figure 2).

**Figure 3:**
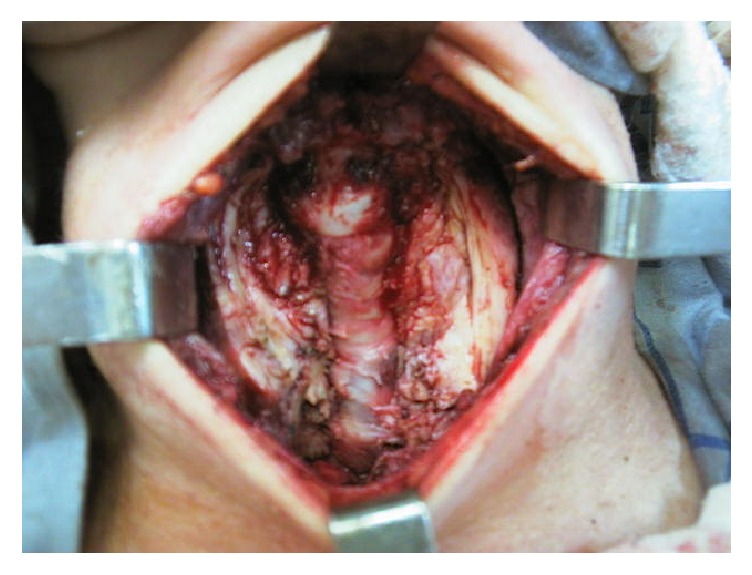
Intraoperative aspect of the tracheal decompression under an intense fibrotic thyroiditis.

The patient underwent surgical decompression of the airway (Figure 3), protection tracheotomy, and gastrostomy for nutritional intake. An extremely fibrotic and hard gland with tracheal infiltration was found. The intraoperative frozen section exam confirmed the diagnosis of Riedel's thyroiditis.

**Figure 4:**
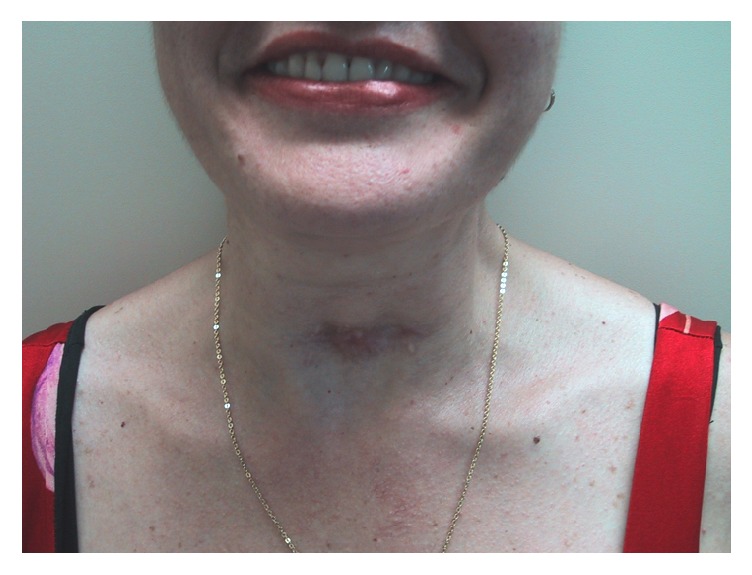
Aspect 2 years after surgery.

The follow-up was satisfactory using prednisolone 40 mg/day. Her symptoms improved 5 months after surgery and the feeding intake was exclusively oral. Her weight recovered and the tracheal cannula was removed 5 months after the tracheotomy due to the improvement of the vocal folds motion. The prednisolone was discontinued after 2 years and the evolution has been excellent, nowadays only using levothyroxine (Figure 4).

**Figure 5:**
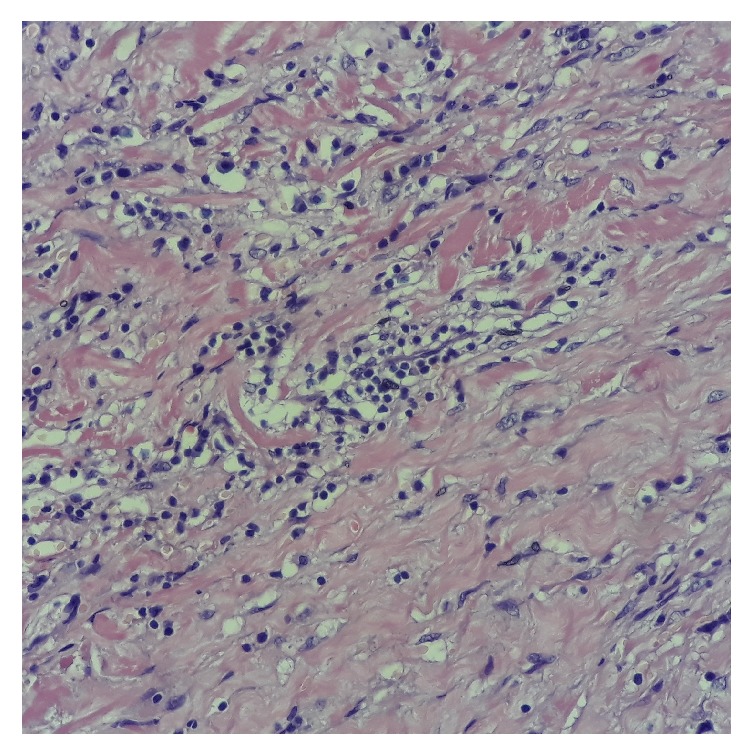
Hematoxylin and eosin staining of the thyroid lesions revealed lymphoplasmacytic infiltration, severe fibrosis, and phlebitis.

**Figure 6:**
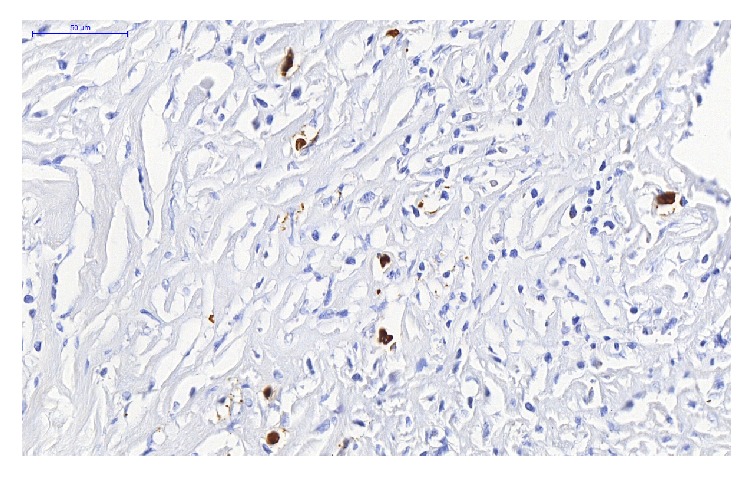
IgG4 immunostaining revealed the presence of IgG4-positive plasma cells.

We were unable to perform the blood dosage of IgG4 and IgG at the time of the active disease. The hematoxylin and eosin staining of the thyroid lesions revealed lymphoplasmacytic infiltration, severe fibrosis, and phlebitis (Figure 5). The IgG4 immunostaining revealed the total number of IgG4-positive plasma cells 12/HPF (Figure 6), and the IgG4/IgG ratio was 25%.

## 3. Discussion

Riedel's thyroiditis is a fibrosclerosing disease whose anatomopathologic aspect is a lymphocytic, plasmocytic, and histiocytic cell infiltration [1, 3]. This is a typical autoimmune response, with mature and hyalinizing fibrosis, similar to that found in Hashimoto's thyroiditis in its final or fibrotic status. The gland is extremely hard and infiltrates into the surrounding tissues. As a result, this condition can be mistaken for malignant neoplasm.

Dyspnea and hoarseness can be found due to tracheal compression or recurrent laryngeal nerve involvement. Furthermore, dysphagia can be the result of pharyngoesophageal involvement and compression [6]. The thyroid function depends on the extension of the replacement of normal tissue by fibrosis. Thus, 64% of the patients present hypothyroidism, whereas 32% are euthyroidism and 4% hyperthyroidism [1]. Hypoparathyroidism can be observed. However, it is reversible under the administration of prednisolone. Anyway, it was not found in our case.

Open surgical biopsy during the cervicotomy is mandatory in order to confirm the diagnosis. Isthmusectomy is enough in order to perform the diagnosis. Broad resections should be avoided due to the risk of injuring the surrounding structures.

An exuberant clinical status with consumption and fast evolution can suggest the undifferentiated carcinoma, such as in the present case reported. The hypothesis of Riedel's thyroiditis was considered because of the FNAB exam. The operation was technically hard to perform, since there was a lack of dissection plane between the thyroid and the trachea, with high risk of injuries to tissues. The patient's weight recovered fast, after starting the use of corticoid. If this treatment is not efficient, other drugs could be employed, such as tamoxifen [2]. Low doses of external beam radiotherapy are also an alternative approach. Spontaneous resolution has also been described [1].

In spite of being uncommon, Riedel's thyroiditis should be considered a differential diagnosis among the anterior neck masses with fast growth. Severe dysphagia has not been reported as a main symptom.

In order to characterize the relationship between RT and IgG4, we performed IgG4 and IgG staining. The total number of IgG4-positive plasma cells was more than 10/HPF, but the IgG4/IgG ratio was less than 40%. IgG4 immunostaining is important for the diagnosis, especially when serum IgG4 is not elevated. Although IgG4 plasma cells per high-power field has an acceptable specificity, an IgG4/IgG plasma cell ratio of greater than 40% is considered more valuable, as some inflammatory lesions have high IgG4 plasma cells [12]. For this case with exuberant symptoms, our expectation would be a larger number of IgG4-positive plasma cells and a larger IgG4/IgG ratio.

There is no study associating intensity of the symptoms with the count of inflammatory plasma cells in Riedel's thyroiditis, but in the spectrum of IgG4 related diseases Deshpande et al. [13] studied a group of patients with Fibrous Variant of a Hashimoto Thyroiditis (FVHT) and compared them with typical Hashimoto Thyroiditis (HT) patients, finding that FVHT patients have more hypothyroidism, a higher mean IgG4-positive cell count in affected thyroid tissue, and a higher IgG4/IgG ratio than typical HT patients.

As to this case, our impression is that there is no relation between the intensity of the thyroiditis, the total number of IgG4-positive plasma cells and the IgG4/IgG ratio. The guidelines proposed do not supplant careful clinicopathological correlation and sound clinical judgment [10].

Since RT is such a rare disease, immunohistochemical analyses have only been performed in limited cases and long-term investigations are further needed.
